# Crystal structure and Hirshfeld surface analysis of 2,5-di­bromo­terephthalic acid ethyl­ene glycol monosolvate

**DOI:** 10.1107/S2056989019010260

**Published:** 2019-07-23

**Authors:** Kenika Khotchasanthong, Siripak Jittirattanakun, Suwadee Jiajaroen, Chatphorn Theppitak, Kittipong Chainok

**Affiliations:** a Materials and Textile Technology, Faculty of Science and Technology, Thammasat University, Khlong Luang, Pathum Thani, 12121, Thailand; b Division of Chemistry, Faculty of Science and Technology, Thammasat University, Khlong Luang, Pathum Thani, 12121, Thailand

**Keywords:** crystal structure, halogen bonds, hydrogen bonds, solvate

## Abstract

The title compound, C_8_H_4_Br_2_O_4_·C_2_H_6_O_2_, crystallizes with one-half of a 2,5-di­bromo­terephthalic acid (H_2_Br_2_tp) mol­ecule and one-half of an ethyl­ene glycol (EG) mol­ecule in the the asymmetric unit. The whole mol­ecules are generated by application of inversion symmetry. The H_2_Br_2_tp mol­ecule is not planar, with the di­bromo­benzene ring system inclined by a dihedral angle of 18.62 (3)° to the carb­oxy­lic group. In the crystal, the H_2_Br_2_tp and EG mol­ecules are linked into sheets propagating parallel to (01) through O—H⋯O hydrogen bonds, thereby forming *R*_4_^4^ (12) and *R*_4_^4^ (28) graph-set motifs. Br⋯O and weak π–π stacking inter­actions are also observed. Hirshfeld surface analysis was used to confirm the existence of these inter­actions.

## Abstract 2

The crystal structure of the title mol­ecular solvate features O—H⋯O hydrogen bonds, Br⋯O and π–π inter­actions. Hirshfeld surface analysis and fingerprint plots helped to identify the major contributors to the inter­molecular inter­actions.

## Chemical context   

Terephthalic acid and its derivatives are important ligands in the construction of coordination frameworks with high dimensionalities and inter­esting topologies (Li *et al.*, 1999 ▸; Seidel *et al.*, 2011 ▸). They have also been shown to be versatile building blocks in crystal engineering to drive the self-assembly of functional supra­molecular networks through inter­molecular inter­actions such as hydrogen bonds, halogen bonds, and aromatic π–π stacking inter­actions (Lemmerer, 2011 ▸; Karmakar *et al.*, 2014 ▸; Meng *et al.*, 2015 ▸).
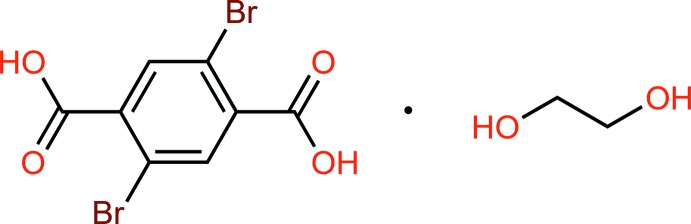


In this study, we present the crystal structure of 2,5-di­bromoterephthalic acid ethyl­ene glycol monosolvate, C_8_H_4_Br_2_O_4_·C_2_H_6_O_2_ or H_2_Br_2_tp·EG, which is a pseudopolymorph of the previously reported compound 2,5-di­bromo­terephthalic acid dihydrate (Song *et al.*, 2008 ▸).

## Structural commentary   

**Figure 1:**
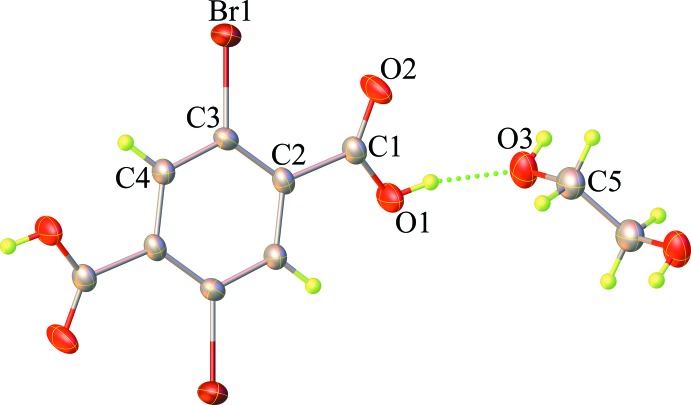
The structures of the mol­ecular components in the title compound with displacement ellipsoids drawn at the 50% probability level. The O—H⋯O hydrogen bond is shown by a dashed line.

The structures of the mol­ecular components in the title compound are shown in Fig. 1 ▸. The asymmetric unit contains one-half of a H_2_Br_2_tp mol­ecule and one-half of an EG mol­ecule. Both mol­ecules are generated by application of inversion symmetry. The H_2_Br_2_tp mol­ecule is not planar. Its di­bromo­benzene ring system (r.m.s. deviation = 0.006 Å) makes a dihedral angle of 18.62 (3)° with the carboxylic group (r.m.s. deviation = 0.013 Å). As a result of symmetry restrictions, the EG mol­ecule adopts an *anti*-conformation with an O3—C5—C5^i^—O3^i^ torsion angle of 180° [symmetry code: (i) 2 − *x*, −*y*, 2 − *z*].

## Supra­molecular features   

**Figure 2:**
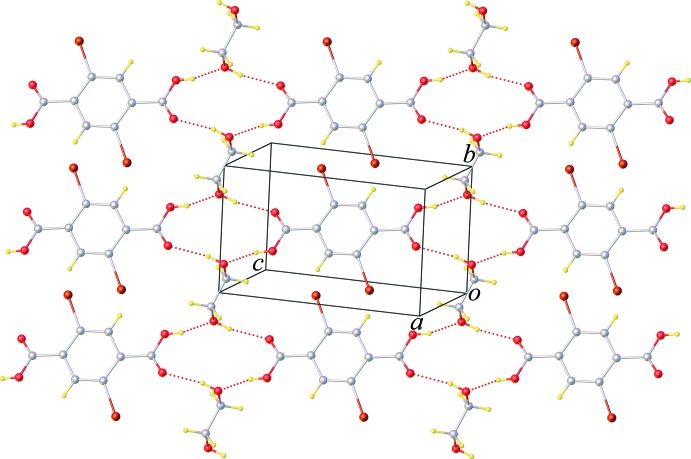
View of a supra­molecular two-dimensional sheet parallel to the (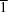
01) direction, enclosing 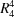 (12) and 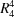 (28) graph-set motifs, sustained by O—H⋯O hydrogen bonds (dashed lines).

**Figure 3:**
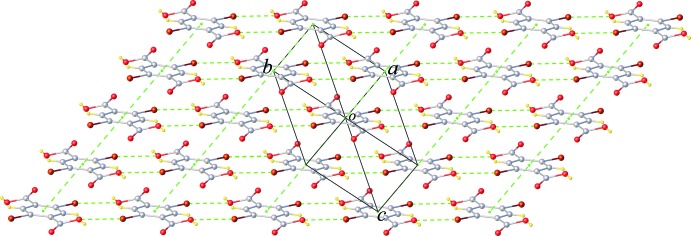
View along [111] of a supra­molecular sheet sustained by Br⋯O halogen bonding and π–π stacking inter­actions (dashed lines).

In the crystal, the H_2_Br_2_tp and EG mol­ecules are linked by strong-to-medium O—H⋯O hydrogen bonds between carb­oxy­lic acid and alcohol OH functions (Table 1 ▸), enclosing 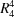 (12) and 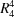 (28) graph-set motifs and forming sheets parallel to the (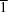
01) plane; Fig. 2 ▸. Br⋯O halogen bonding [Br⋯O = 3.2536 (4) Å; C—Br⋯O = 157.7 (3)°] and weak π–π stacking inter­actions [centroid-to-centroid distance = 4.283 (5) Å] are also observed (Fig. 3 ▸). The combination of these inter­molecular inter­actions results in the formation of a three-dimensional supra­molecular network.

## Hirshfeld surface analysis   

**Figure 4:**
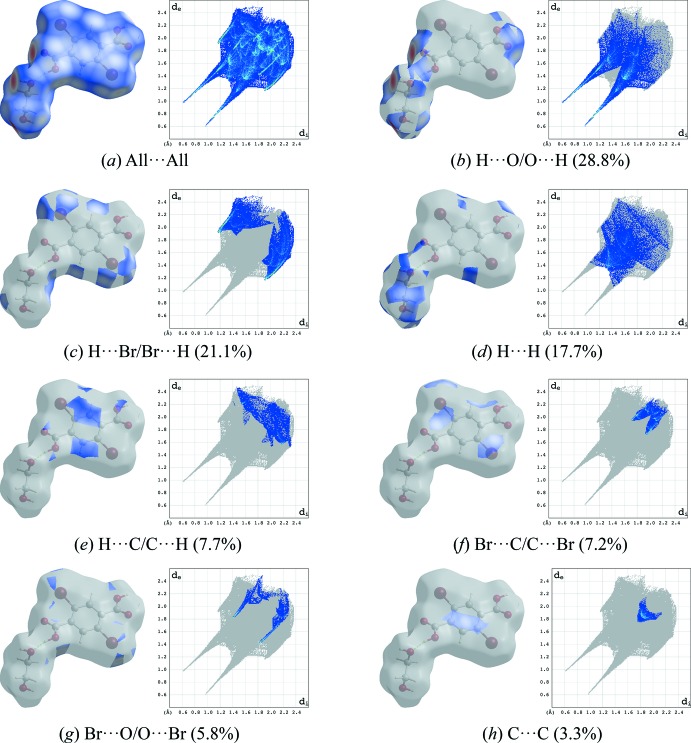
Two-dimensional fingerprint plots of the title compound, showing (*a*) all inter­actions, and delineated into (*b*) H⋯O/O⋯H, (*c*) H⋯Br/Br⋯H, (*d*) H⋯H, (*e*) H⋯C/C⋯H, (*f*) H⋯O/O⋯H, (*g*) Br⋯O/O⋯Br, and (*h*) C⋯C inter­actions [*d*_e_ and *d*_i_ represent the distances from a point on the Hirshfeld surface to the nearest atoms outside (external) and inside (inter­nal) the surface, respectively].

Hirshfeld surfaces (McKinnon *et al.*, 2007 ▸) and their associated two-dimensional fingerprint plots (Spackman & McKinnon, 2002 ▸) were used to qu­antify the various inter­molecular inter­actions, and were generated using *CrystalExplorer17* (Turner *et al.*, 2017 ▸). The shorter and longer contacts are indicated as red and blue spots on the Hirshfeld surfaces, and contacts with distances equal to the sum of the van der Waals radii are represented as white spots. Hirshfeld surfaces of the title compound mapped over the normalized distance, *d*_norm_, using a standard surface resolution with a fixed colour scale of −0.7877 (red) to 0.9385 a.u. (blue) and the two-dimensional fingerprint plots are illustrated in Fig. 4 ▸. The dominant inter­actions between H and O atoms, corresponding to the discussed O—H⋯O hydrogen bonds, can be clearly be seen as red spots on the Hirshfeld surface. The faint-red spot visible on the *d*_norm_ surface can be assigned to Br⋯O contacts. Analysis of the two-dimensional fingerprint plots reveals that the H⋯O/O⋯H (28.8%) contacts are the dominant contributors to the Hirshfeld surface. The contribution of the Br⋯H/H⋯Br contacts is 22.1%, whereas Br⋯Br contacts are negligible (0.9%). Other contacts *viz*. H⋯H (17.7%), H⋯C/C⋯H (7.7%), Br⋯C/C⋯Br (7.2%), Br⋯O/O⋯Br (5.8%), C⋯O/O⋯C (4.5%), C⋯C (3.3%) and O⋯O (2.2%) also make significant contributions to the Hirshfeld surface.

## Database survey   

A search of the Cambridge Structural Database (Version 5.40, latest update May 2019; Groom *et al.*, 2016 ▸) for the H_2_Br_2_tp entity resulted in just two matches. In the structure of the pseudopolymorphic H_2_Br_2_tp dihydrate (CSD refcode POFROS; Song *et al.*, 2008 ▸), the H_2_Br_2_tp mol­ecules are connected through water mol­ecules by O—H⋯O hydrogen bonds, forming a three-dimensional supra­molecular network. In the structure of bis­{*N*-[1-(pyridin-2-yl-κ*N*)ethyl­idene]pyridine-4-carbohydrazonato-κ^2^ *N*′,*O*}nickel(II)–2,5-di­bromo­terephthalic acid (OBOJEX; Nakanishi & Sato, 2017 ▸), the H_2_Br_2_tp mol­ecules form hydrogen-bonded zigzag chains with the complex mol­ecules. The packing is further consolidated by π–π stacking and Br⋯Br halogen bonding.

## Synthesis and crystallization   

H_2_Br_2_tp and EG were purchased from commercial sources and used as received. A solution of H_2_Br_2_tp (0.020 g) in 5 ml of EG was heated (333 K) to reflux for 15 min. The reaction solution was held for 2–3 h and colourless block-shaped crystals suitable for single-crystal X-ray diffraction analysis were obtained.

## Refinement   

Crystal data, data collection and structure refinement details are summarized in Table 2 ▸. The carbon-bound H atoms were placed in geometrically calculated positions and refined as riding with C—H = 0.93 Å for aromatic and C—H = 0.97 Å for methyl­ene hydrogen atoms with *U*_iso_(H) = 1.2*U*_eq_(C). The H atoms bound to O atoms were located from difference-Fourier maps but were refined with distance restraints of O—H = 0.82 ± 0.02 Å and *U*_iso_(H) = 1.5*U*_eq_(O).

## Supplementary Material

Crystal structure: contains datablock(s) I. DOI: 10.1107/S2056989019010260/wm5512sup1.cif

Supporting information file. DOI: 10.1107/S2056989019010260/wm5512Isup3.cdx

Structure factors: contains datablock(s) I. DOI: 10.1107/S2056989019010260/wm5512Isup4.hkl

CCDC reference: 1941436

Additional supporting information:  crystallographic information; 3D view; checkCIF report

## Figures and Tables

**Table 1:** Hydrogen-bond geometry (Å, °)

*D*—H⋯*A*	*D*—H	H⋯*A*	*D*⋯*A*	*D*—H⋯*A*
O1—H1⋯O3	0.83 (1)	1.75 (1)	2.559 (3)	165 (4)
O3—H3⋯O2^i^	0.82 (1)	1.97 (1)	2.767 (3)	166 (4)

Symmetry code: (i) 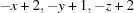.

**Table 2:** Experimental details

Crystal data	
Chemical formula	C_8_H_4_Br_2_O_4_·C_2_H_6_O_2_
*M*_r_	386.00
Crystal system, space group	Triclinic, *P* 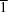
Temperature (K)	296
*a*, *b*, *c* (Å)	4.2823 (6), 6.2607 (9), 11.5497 (17)
α, β, γ (°)	96.701 (5), 93.930 (5), 90.575 (5)
*V* (Å^3^)	306.76 (8)
*Z*	1
Radiation type	Mo *K*α
μ (mm^−1^)	6.62
Crystal size (mm)	0.20 × 0.20 × 0.16

Data collection	
Diffractometer	Bruker D8 QUEST CMOS PHOTON II
Absorption correction	Multi-scan (*SADABS*; Bruker, 2016 ▸)
*T*_min_, *T*_max_	0.576, 0.747
No. of measured, independent and observed [*I* > 2σ(*I*)] reflections	9075, 1208, 1076
*R*_int_	0.052
(sin θ/λ)_max_ (Å^−1^)	0.617

Refinement	
*R*[*F* ^2^ > 2σ(*F* ^2^)], *wR*(*F* ^2^), *S*	0.023, 0.052, 1.05
No. of reflections	1208
No. of parameters	91
No. of restraints	2
H-atom treatment	H atoms treated by a mixture of independent and constrained refinement
Δρ_max_, Δρ_min_ (e Å^−3^)	0.34, −0.28

Computer programs: *APEX3* and *SAINT* (Bruker, 2016 ▸), *SHELXT* (Sheldrick, 2015*a* ▸), *SHELXL2014* (Sheldrick, 2015*b* ▸ and *OLEX2* (Dolomanov *et al.*, 2009 ▸).

## supplementary crystallographic information

### Crystal data

**Table:**

C_8_H_4_Br_2_O_4_·C_2_H_6_O_2_	*Z* = 1
*M**_r_* = 386.00	*F*(000) = 188
Triclinic, *P*1	*D*_x_ = 2.089 Mg m^−^^3^
*a* = 4.2823 (6) Å	Mo *K*α radiation, λ = 0.71073 Å
*b* = 6.2607 (9) Å	Cell parameters from 5153 reflections
*c* = 11.5497 (17) Å	θ = 3.3–28.8°
α = 96.701 (5)°	µ = 6.62 mm^−^^1^
β = 93.930 (5)°	*T* = 296 K
γ = 90.575 (5)°	Block, light colourless
*V* = 306.76 (8) Å^3^	0.20 × 0.20 × 0.16 mm

### Data collection

**Table:**

Bruker D8 QUEST CMOS PHOTON II diffractometer	1208 independent reflections
Radiation source: sealed x-ray tube, Micro focus tube	1076 reflections with *I* > 2σ(*I*)
Graphite monochromator	*R*_int_ = 0.052
Detector resolution: 7.39 pixels mm^-1^	θ_max_ = 26.0°, θ_min_ = 3.3°
ω and φ scans	*h* = −5→5
Absorption correction: multi-scan (*SADABS*; Bruker, 2016)	*k* = −7→7
*T*_min_ = 0.576, *T*_max_ = 0.747	*l* = −14→14
9075 measured reflections	

### Refinement

**Table:**

Refinement on *F*^2^	Hydrogen site location: mixed
Least-squares matrix: full	H atoms treated by a mixture of independent and constrained refinement
*R*[*F*^2^ > 2σ(*F*^2^)] = 0.023	*w* = 1/[σ^2^(*F*_o_^2^) + (0.0175*P*)^2^ + 0.2228*P*] where *P* = (*F*_o_^2^ + 2*F*_c_^2^)/3
*wR*(*F*^2^) = 0.052	(Δ/σ)_max_ < 0.001
*S* = 1.05	Δρ_max_ = 0.34 e Å^−^^3^
1208 reflections	Δρ_min_ = −0.28 e Å^−^^3^
91 parameters	Extinction correction: SHELXL-2018/3 (Sheldrick 2018), Fc^*^=kFc[1+0.001xFc^2^λ^3^/sin(2θ)]^-1/4^
2 restraints	Extinction coefficient: 0.034 (3)
Primary atom site location: dual	

### Special details

**Table:**

Geometry. All esds (except the esd in the dihedral angle between two l.s. planes) are estimated using the full covariance matrix. The cell esds are taken into account individually in the estimation of esds in distances, angles and torsion angles; correlations between esds in cell parameters are only used when they are defined by crystal symmetry. An approximate (isotropic) treatment of cell esds is used for estimating esds involving l.s. planes.

### Fractional atomic coordinates and isotropic or equivalent isotropic displacement parameters (Å^2^)

**Table:**

	*x*	*y*	*z*	*U*_iso_*/*U*_eq_	
Br1	0.28840 (7)	0.90781 (4)	0.67985 (2)	0.03714 (15)	
O1	0.9576 (5)	0.3651 (3)	0.75182 (16)	0.0399 (5)	
H1	1.013 (9)	0.352 (6)	0.8205 (14)	0.071 (12)*	
O2	0.6124 (5)	0.5932 (3)	0.82669 (15)	0.0430 (5)	
O3	1.1223 (5)	0.2556 (3)	0.95346 (17)	0.0415 (5)	
H3	1.205 (8)	0.320 (5)	1.0136 (19)	0.065 (12)*	
C1	0.7228 (6)	0.4945 (4)	0.7436 (2)	0.0263 (5)	
C2	0.6046 (5)	0.5048 (4)	0.6187 (2)	0.0231 (5)	
C3	0.4170 (6)	0.6668 (4)	0.5809 (2)	0.0244 (5)	
C4	0.3155 (6)	0.6610 (4)	0.4647 (2)	0.0261 (5)	
H4	0.190106	0.770860	0.441344	0.031*	
C5	0.9128 (7)	0.0972 (4)	0.9857 (2)	0.0371 (6)	
H5A	0.756819	0.056821	0.921709	0.044*	
H5B	0.805174	0.156980	1.052949	0.044*	

### Atomic displacement parameters (Å^2^)

**Table:**

	*U*^11^	*U*^22^	*U*^33^	*U*^12^	*U*^13^	*U*^23^
Br1	0.0530 (2)	0.03148 (18)	0.02537 (18)	0.01196 (12)	0.00214 (11)	−0.00380 (10)
O1	0.0505 (12)	0.0469 (12)	0.0215 (10)	0.0198 (9)	−0.0049 (9)	0.0034 (9)
O2	0.0507 (12)	0.0585 (13)	0.0175 (9)	0.0199 (10)	−0.0031 (8)	−0.0034 (9)
O3	0.0535 (13)	0.0409 (11)	0.0290 (11)	−0.0047 (10)	−0.0110 (10)	0.0079 (9)
C1	0.0292 (13)	0.0285 (13)	0.0206 (12)	−0.0021 (10)	−0.0026 (10)	0.0029 (10)
C2	0.0261 (12)	0.0261 (12)	0.0170 (11)	−0.0032 (10)	−0.0007 (9)	0.0037 (9)
C3	0.0299 (13)	0.0239 (12)	0.0189 (11)	0.0015 (10)	0.0031 (10)	−0.0006 (9)
C4	0.0307 (13)	0.0272 (12)	0.0204 (12)	0.0050 (10)	−0.0011 (10)	0.0044 (10)
C5	0.0386 (15)	0.0414 (15)	0.0310 (14)	0.0065 (12)	−0.0030 (12)	0.0062 (12)

### Geometric parameters (Å, º)

**Table:**

Br1—C3	1.894 (2)	C2—C3	1.392 (3)
O1—H1	0.825 (10)	C2—C4^i^	1.394 (3)
O1—C1	1.303 (3)	C3—C4	1.377 (3)
O2—C1	1.207 (3)	C4—H4	0.9300
O3—H3	0.817 (10)	C5—C5^ii^	1.493 (5)
O3—C5	1.428 (4)	C5—H5A	0.9700
C1—C2	1.504 (3)	C5—H5B	0.9700
			
C1—O1—H1	112 (3)	C4—C3—C2	120.5 (2)
C5—O3—H3	108 (3)	C2^i^—C4—H4	119.1
O1—C1—C2	112.1 (2)	C3—C4—C2^i^	121.9 (2)
O2—C1—O1	123.7 (2)	C3—C4—H4	119.1
O2—C1—C2	124.1 (2)	O3—C5—C5^ii^	110.6 (3)
C3—C2—C1	124.6 (2)	O3—C5—H5A	109.5
C3—C2—C4^i^	117.6 (2)	O3—C5—H5B	109.5
C4^i^—C2—C1	117.8 (2)	C5^ii^—C5—H5A	109.5
C2—C3—Br1	124.11 (18)	C5^ii^—C5—H5B	109.5
C4—C3—Br1	115.39 (18)	H5A—C5—H5B	108.1
			
O3—C5—C5^ii^—O3^ii^	180.000 (1)		

Symmetry codes: (i) −*x*+1, −*y*+1, −*z*+1; (ii) −*x*+2, −*y*, −*z*+2.

### Hydrogen-bond geometry (Å, º)

**Table:**

*D*—H···*A*	*D*—H	H···*A*	*D*···*A*	*D*—H···*A*
O1—H1···O3	0.83 (1)	1.75 (1)	2.559 (3)	165 (4)
O3—H3···O2^iii^	0.82 (1)	1.97 (1)	2.767 (3)	166 (4)

Symmetry code: (iii) −*x*+2, −*y*+1, −*z*+2.
